# Elevated serum matrix metalloproteinase 9 concentration predicts the presence of colorectal neoplasia in symptomatic patients

**DOI:** 10.1038/sj.bjc.6604338

**Published:** 2008-04-15

**Authors:** D P Belobrajdic, L J Cosgrove, A Phatak

**Affiliations:** 1 CSIRO Preventative Health Flagship, Adelaide, Australia; 2 CSIRO Molecular and Health Technologies, Kintore Avenue, Adelaide, SA 5000, Australia; 3 CSIRO Mathematical and Information Sciences, Wembley, WA 6913, Australia

**Sir**,

We have read with great interest the article by Hurst *et al* (2007). In their study, they make prospective predictions—that is, without knowing the definitive diagnosis—of underlying colorectal neoplasia (their Figure 1) based on serum MMP-9 (sMMP-9) concentration. The criterion they use to prospectively classify patients is the ratio of measured to predicted sMMP-9 concentration, where a ratio greater than one indicates neoplasia and less than or equal to one indicates non-neoplasia. The model was fitted using only normal, disease-free individuals and then, as far as we are able to ascertain, applied to all (diseased and disease-free) individuals.

We recognise that one of the strengths of this study is that blood was collected before diagnosis of disease, thereby, removing a potential source of bias. Nevertheless, it is not clear to us why prospective prediction was carried out and reported, especially when the authors subsequently carry out a regression analysis that yields much better sensitivity and specificity. We also do not understand why an observed to predicted ratio of one ought to provide an optimal cutoff to prospectively distinguish between non-neoplasia and neoplasia. Finally, the R^2^ value of 0.027 quoted by the authors is extremely small, and unless it is a misprint, indicates that the data do not support the assertion of a relationship between sMMP-9 concentration and patient age. Indeed, it is straightforward to show that in the absence of any relationship, the ‘predicted’ value is simply the mean sMMP-9 concentration of the normal, disease-free observations and is, therefore, the cutoff point for prospectively predicting non-neoplasia or neoplasia.

Lack of space may have precluded its inclusion, but a plot of sMMP-9 concentration against age would have assisted readers in judging whether fitting a model relating concentration and age was justified. Moreover, whether or not concentration depends on other covariates, it would be useful to display the measured concentrations of sMMP-9—as boxplots, for example— for all subgroups in Figure 1 of their paper.

In a burgeoning field, such as biomarker discovery and validation, where considerable effort is required to generate reliable data—as Hurst *et al* have demonstrated here—and where well-known and novel statistical methods are employed for data analysis, authors should be encouraged to publish raw data in web-based appendices to allow others to independently reproduce their data analyses (Altman and Cates, 2001; Hutchon, 2001). We note that policy of *British Journal of Cancer* requires authors to make available to readers materials that are not readily available from commercial suppliers, and this requirement could be extended to data, which could be made available as supplementary online material.
